# Some Scientific Aspects of Insanity

**Published:** 1892-02-06

**Authors:** 


### Some Scientific Aspects of Insanity.

### XI.—EPILEPTICS.

The ordinary forms of insanity which are met with in association with paralysis and convulsions are epilepsy and general paralysis.

1. *Epilepsy.*—There are epileptics who never become insane, and it does not follow that because a person takes epileptic fits he will ultimately become a lunatic. The occurrence of insanity in epilepsy does not depend either upon the character or the number of the fits. A patient may be quite demented who only takes fits at rare intervals, while another patient may take daily, or weekly, or monthly fits, and yet in the interval between the fits be comparatively sane. The epileptic insane usually met with in asylums are a very distinctive and characteristic class. It may be interesting to note a few of their peculiarities. They are very fond of each other's society, and usually sit together, converse together, and are jealous of the intrusions of other patients. They are very religious, at any rate so far as the outward forms of religion are concerned. Most of them read their Bibles and pray, perhaps, ostentatiously. They are extremely irritable, are easily roused into the most uncontrollable outbursts of passion upon very slight provocation. They all manifest a remarkable softness and suavity of manner, a desire to be agreeable and obliging, and a politeness that is often irresistible. The management of people whose mental disposition is so peculiarly irritable requires on the part of those who are entrusted with their care the greatest possible amount of tact. At the same time the great similarity of the mental constitution of epileptics renders it easier to deal with them as a class. It should be remembered that before, in the interval between, and immediately after taking fits, the greatest caution is required on account of their violence and explosiveness of temper. It will very generally be observed that there are two kinds of insanity met with in the epileptic inmates of asylums, and sometimes during the period of taking fits both kinds are seen in the same patient. There is (1) the joyous, hilarious singing mania that immediately precedes the fit; and (2) the moody, furious, unconscious destructive mania that follows it. Many epileptic inmates of asylums are in the intervals between their convulsive seizures to all intents and purposes intellectually sane, and they require, therefore, in all respects to be treated as such. Epileptic fits in large asylums are matters of daily and hourly occurrence, and on account of their frequency they receive less attention than they deserve. If every attendant who has charge of an epileptic patient were to record with as much exactitude as possible the various and varying particulars of each seizure, our knowledge might be very much extended. An epileptic fit, like everything else, requires a certain amount of knowledge of it before accurate observations can be recorded. How very few people, for instance, know the average duration of an epileptic fit. How few could give an account of its different stages. In order that your attention may be directed to this important manifestation of nervous disease, I shall briefly describe one of these seizures.

Many epileptics have what is termed a "warning" of the approach of a fit. The "warning" may take the form of increased irritability of temper, a sensation creeping up a limb, a peculiar feeling in the stomach which chokes the patient, an hallucination of one of the senses, and many other forms. The first stage of the fit is characterised by paleness of the face, or by the following symptoms: (1) paleness of the face; (2) the characteristic epileptic cry; (3) tonic contraction of the muscles of the body; (4) fall. The first stage, then, is characterised by tonic spasm of the arteries of the head and face causing paleness, of the muscles of the throat causing the cry, and of the muscles of the trunk and limbs, bringing about loss of equilibrium and consequent fall. This stage lasts in all from ten to twenty seconds. It is called the tonic stage.

The second stage is called the clonic stage, because the patient's muscles are in a state of clonic contraction. Tonic contraction means a steady, uninterrupted contraction, just as if one were to take into their hands a small india-rubber ball, and slowly and gradually compress it until all the air was driven out, and the ball remained flattened in the hand. Clonic contraction, on the other hand, is where there is interrupted contraction of muscles, just as if the same india-rubber ball were taken into the palm of the hand and compressed in a jerky, spasmodic manner. In the second stage of the epileptic fit there is, first, a clonic contraction of all the muscles of the body; second, sweating, with increase of temperature; third, a purple, livid colour of the face, and frothing at the mouth.

The third stage is the stage of coma—that is, of deep, unconscious sleep, in which the patient breathes heavily and is oblivious to all that goes on around him. The first two stages constitute the convulsive seizure proper, and they very seldom exceed ninety seconds in duration. The patient may sleep for an indefinite period after the fit.

The sleep following an epileptic fit is generally restorative, and it is often worth while to take a great deal of trouble to secure to the patient a sufficient amount of sleep, as in this way in some cases the violent insanity that often immediately succeeds the seizure can be averted. The patient should, therefore, in such cases be carefully and gently removed to a bed-room specially prepared, or, if not prepared, all dangerous or breakable articles should be removed; the patient should be carefully laid upon the bed, covered over with a rug, and the windows should be darkened. The most absolute silence should at the same time be observed. Where such a course is not practicable, or not required, the attendant should by no means leave the patient lying on the floor. As soon as the advent of a fit is observed, all clothing about the neck and chest should be loosened so as to render danger from asphyxia as slight as possible. He should then be raised and placed upon one of those suitable couches which are now provided in every modern asylum. It should not be permitted to other patients to suppose that their fellow sufferers are in their misfortune not properly cared for and respected by the attendants. It should never be forgotten by the guardian that during a fit the epileptic patient passes urine involuntarily.

There is a drug which has had the most beneficial effect in relieving the irritability, in reducing the number of fits, and in modifying the violence of the mania which follows the seizures. The drug is called bromide of potassium. I have mentioned it for the purpose of delivering a note of warning against its too frequent or intemperate use. It has been found by wide experience that this drug, when administered in small doses with unfailing regularity, invariably produces a modification of the symptoms of the disease. It is so commonly used in asylums, and in itself so apparently harmless, that it has, I fear, become a not uncommon habit on the part of attendants to administer larger doses, and if the patient is specially irritable more frequent doses than what is prescribed by the medical officers. No greater mistake could possibly be committed, for the increased and irregular administration does great harm by reducing the bodily health, adding to the excitability of the patient, and increasing the frequency of fits.

There is another danger to which the chronic epileptic are frequently subjected. For a reason which is not satisfactorily explained the times at which fits frequently occur are immediately before or after the patient has begun to take food. It is, therefore, necessary that the attendants should be prepared for the emergency of choking and be able to remove from the mouth as much unmasticated food as they can reach with their fingers.

